# Investigating Potential Anti-Bacterial Natural Products Based on Ayurvedic Formulae Using Supervised Network Analysis and Machine Learning Approaches

**DOI:** 10.3390/ph18020192

**Published:** 2025-01-30

**Authors:** Pei Gao, Ahmad Kamal Nasution, Naoaki Ono, Shigehiko Kanaya, Md. Altaf-Ul-Amin

**Affiliations:** Graduate School of Science and Technology, Nara Institute of Science and Technology (NAIST), Ikoma 630-0101, Nara, Japan; gao.pei.gi3@is.naist.jp (P.G.); kamal.nasution@naist.ac.jp (A.K.N.); nono@is.naist.jp (N.O.); skanaya@is.naist.jp (S.K.)

**Keywords:** Ayurvedic, medicinal plants, network analysis, machine learning

## Abstract

**Objectives**: This study implements a multi-dimensional methodology to systematically identify potential natural antibiotics derived from the medicinal plants utilized in Ayurvedic practices. **Methods**: Two primary analytical techniques are employed to explore the antibiotic potential of the medicinal plants. The initial approach utilizes a supervised network analysis, which involves the application of distance measurement algorithms to scrutinize the interconnectivity and relational patterns within the network derived from Ayurvedic formulae. **Results**: 39 candidate plants with potential natural antibiotic properties were identified. The second approach leverages advanced machine learning techniques, particularly focusing on feature extraction and pattern recognition. This approach yielded a list of 32 plants exhibiting characteristics indicative of natural antibiotics. A key finding of this research is the identification of 17 plants that were consistently recognized by both analytical methods. These plants are well-documented in existing literature for their antibacterial properties, either directly or through their bioactive compounds, which suggests a strong validation of the study’s methodology. By synergizing network analysis with machine learning, this study provides a rigorous and multi-faceted examination of Ayurvedic medicinal plants, significantly contributing to the identification of natural antibiotic candidates. **Conclusions**: This research not only reinforces the potential of traditional medicine as a source for new therapeutics but also demonstrates the effectiveness of combining classical and contemporary analytical techniques to explore complex biological datasets.

## 1. Introduction

The therapeutic efficacy of existing antibiotics for common infectious diseases is progressively diminishing, largely due to the widespread and often inappropriate use of these drugs [1]. This indiscriminate antibiotic use is a fundamental factor contributing to the reduced effectiveness in combating pathogenic bacteria, leading to the emergence of multidrug-resistant (MDR) bacteria in clinical settings [2]. Despite concerted efforts by researchers and practitioners to develop novel antibiotics, the pace of new antibiotic discovery is insufficient to keep up with the rapid proliferation of MDR bacteria [3,4]. The development process is particularly time-consuming and fraught with challenges, including the necessity to conduct extensive large-scale animal testing. These tests are often labor-intensive, costly, and may yield unsatisfactory results, frequently leading to the rediscovery of antibiotics with similar modes of action rather than truly novel therapeutic agents [5]. This scenario underscores the urgent need for innovative and reliable methodologies to accelerate the discovery of new antibiotics and the design of novel drugs.

Conventional methods in antibiotic discovery have traditionally focused on high-throughput screening of synthetic compound libraries [6]. However, the vast chemical space they encompass presents significant challenges. The process of aimlessly traversing this expansive chemical landscape often lacks a targeted approach to the identification of compounds with therapeutic potential, leading to an increased consumption of resources in the validation phase [7,8]. Given these constraints, researchers have increasingly sought alternative sources that could provide more promising sample spaces for antibiotic discovery. In recent years, medicinal plants have emerged as a compelling source in the field of new drug design. The diverse bioactive compounds found in medicinal plants offer a wealth of potential candidates with inherent medicinal value [9]. This has led to a growing interest within the pharmaceutical community, as these natural products present a more targeted and potentially fruitful avenue for the discovery of novel antibiotics [10].

Medicinal plants refer to preparations derived from various parts of plants, including leaves, flowers, roots, and other components, each with distinct medicinal applications [11]. The preparation of these herbal remedies involves diverse extraction methods tailored to the specific chemical constituents present in the plant material [12]. Over the past few decades, herbal medicines have gained significant global popularity, largely due to their perceived superior efficacy and lower incidence of side effects compared to conventional pharmaceuticals [13]. As a result, approximately 80% of the global population currently relies on herbal medicines for their healthcare needs [14]. This burgeoning market reflects the increasing recognition and adoption of herbal medicines as a viable alternative or complement to conventional drug therapies, driven by the growing consumer preference for natural and holistic health solutions.

Ayurveda, one of the world’s oldest holistic healing systems, originated in India over 3000 years ago and remains deeply rooted in Indian culture and practices [15]. The Ayurvedic system of medicine relies heavily on the use of medicinal plants and natural substances, which are carefully selected and prepared according to time-honored practices [16]. These preparations are tailored to the individual’s unique constitution and health needs, with the aim of restoring and maintaining harmony within the body’s internal environment [17]. Ayurvedic medicine encompasses a wide range of therapeutic practices, including dietary regulation, herbal remedies, physical therapies, and spiritual exercises, all designed to promote long-term health and prevent illness [18]. There is a promising potential to bridge the gap between traditional wisdom and modern medical science, thereby enhancing the integration of Ayurvedic principles into drug design.

The formulae of traditional medicine can be regarded as the empirical dataset about combination medication using medicinal plants [19]. Wijaya et al. identified 94 important NPs associated with 12 medicinal efficacy groups based on the mining of the traditional medicine formula, of which many have been confirmed by published literature [20]. This study empowers machine learning to predict natural plant antibiotics based on the Jamu formula (traditional medicine from Indonesia) using the Random Forest approach. Another study quantified the strength of a relationship between TCM (traditional medicine from China) formulae and curative efficacy groups using a graph-based method and introduced a deep network-based feature selection method for finding the potential candidates of new antibiotics design [21]. Hence, we proposed that constructing a relationship between Ayurvedic Formulae, medicinal plants, and antimicrobial efficacy can assist in clarifying the correspondence—that is, help determine the candidates of antimicrobial medicinal plants.

In this study, we employ a systematic and multi-faceted approach to identify potential plant-based natural antibiotics from Ayurvedic formulations. The methodology is structured into four major phases: data acquisition and preprocessing, supervised network clustering, machine learning modeling, and validation. In the initial phase, comprehensive data on Ayurvedic medicinal plants, including their therapeutic applications, was gathered and organized into a tabular format. Each row of the dataset represented an Ayurvedic formula, with features corresponding to the plants included in the formula and a binary class label indicating its effectiveness against bacterial infections. The dataset was then subjected to preprocessing, which involved cleaning and optimizing the data through dimensionality reduction and synthetic data augmentation using SMOTE to balance the dataset. The next phase involved supervised network clustering, where the DPClusO algorithm was applied to cluster the Ayurvedic formulas based on their potential medicinal properties [22]. Minkowski distance was utilized to establish connections between formulas, and a voting mechanism was implemented to identify clusters strongly associated with antibiotic properties. In the machine learning phase, several algorithms were tested, with Random Forest achieving the highest accuracy. This model was further refined by extracting the most significant features, which were used to predict potential plant-based antibiotics. Finally, a rigorous validation process was conducted by reviewing existing scientific literature to confirm the antibacterial properties of the identified plants, ensuring the reliability of the findings.

## 2. Results

### 2.1. Data Preprocessing

We compiled a dataset of Ayurvedic formulas, including detailed information about the medicinal plants used in each formula and their therapeutic applications [23]. The dataset is organized as tabular data, where each row corresponds to a specific Ayurvedic formula. The dataset’s features include the medicinal plants present in each formula and a binary class label. The class label indicates whether the formula is effective in treating bacterial infections, with a value of 1 representing effectiveness (antibiotic class) and 0 indicating ineffectiveness. The dataset used in this study consists of 285 instances and 293 features, with two distinct class labels representing the formulas’ effectiveness in curing bacterial infections.

The dataset was preprocessed using the fundamental tasks, including checking for and removing missing or redundant data. Dimensionality reduction was achieved using Principal Component Analysis (PCA). We selected a number of principal components equivalent to approximately half the original feature set. The final dataset processed by the machine learning protocols consisted of 285 samples (formulae) and 146 principal components. Both the neural network and random forest models were trained and evaluated using this reduced dataset. Columns with too many missing values, low variance, or features with little predictive power were filtered out during preprocessing. Besides, the dataset was augmented by generating new instances of Ayurvedic formulas using the Synthetic Minority Over-sampling Technique (SMOTE) [24].

### 2.2. Supervised Network

After applying the DPClusO algorithm to cluster the Ayurvedic formulas based on their potential medicinal properties, a voting mechanism was implemented to confirm the association of clusters with antibiotic properties based on consensus across multiple formulas. Each plant was found to appear in two to six different formulas within the dataset, indicating their widespread usage and potential significance in traditional medicine systems. These plants were selected for their consistent presence in antibiotic-dominant clusters.

**Figure 1:**
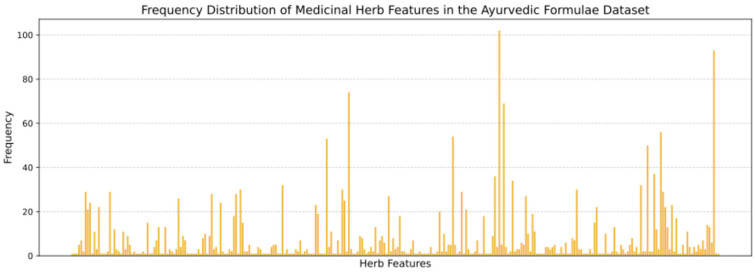
Frequency distribution of Medicinal Herb Features in the Ayurvedic Formulae Dataset.

From the 11 clusters that are dominant for antibiotic properties, we extracted 39 unique plants. The distribution of these plants across the formulae varies, with each plant appearing in two to six different formulas. The detailed results are presented in Table 1 and Figure 1.

### 2.3. Machine Learning for Antimicrobial Phenotype Decision

We conducted experiments using three distinct types of datasets: one obtained after filtering columns to retain only the most relevant features, a second generated using the SMOTE, and a third hybrid dataset created by combining the two aforementioned approaches. While we observed improvements in accuracy across some models in the seven models tested, the magnitude of these improvements was not statistically significant in most cases. As shown in Table 2, the performance indicated that the Random Forest algorithm achieved the highest accuracy at 0.824, followed by Logistic Regression at 0.797, and Gradient Boosting at 0.775.

Given these results, we identified the Random Forest model trained on the SMOTE-filtered dataset as the most suitable approach for the next stage of our analysis. This model’s enhanced performance underscores its potential for effectively predicting plant-based natural antibiotics. A detailed summary of experimental results is provided in Table 3.

Upon achieving the optimal prediction model, we identified the key features of plants contributing to the model’s accuracy. To accomplish this, we leveraged the variable importance attribute of the best Random Forest model. This was implemented using the Random Forest Regressor and Permutation Importance techniques. Based on this approach, we generated a ranked list of potential plants with natural antibiotic properties, ordered according to their significance (weight) in the model. We performed sensitivity analyses using alternative thresholds (between 0.005 and 0.02) and found that the overall trends and key findings remain consistent. Applying the threshold of greater than 0.01, we identified 39 unique potential plant-based natural antibiotics. The medicinal plant and the importance are shown in Table 4.

### 2.4. Overlapping Results

The overlap of results from supervised network analysis and machine learning approaches identifies 17 medicinal plants with antimicrobial properties. These plants are a focus of further investigation into their medicinal components.

*Piper longum*: *Piper longum*, commonly known as “Pippali”, is a well-known medicinal plant in India, Singapore, Malaysia, and other South Asian countries. It has been traditionally used as a carminative, anti-diarrheal, and immunostimulant, as well as in managing conditions such as asthma, insomnia, dementia, epilepsy, diabetes, rheumatoid arthritis, and spleen disorders. Notably, *Piper longum* exhibits potent antibacterial activity [23]. Studies have demonstrated that certain isolates from *Piper longum* are effective against Gram-positive bacteria and show moderate activity against Gram-negative bacteria, highlighting its potential as a natural antimicrobial agent.

*Piper nigrum*: *Piper nigrum* (black pepper) exhibits potent antimicrobial properties due to its primary bioactive compound, piperine. Piperine has demonstrated significant antimicrobial efficacy against both Gram-positive and Gram-negative bacteria. The essential oils of Piper nigrum, particularly rich in compounds like β-caryophyllene and limonene, also contribute to its broad-spectrum antimicrobial action [25,26]. These metabolites disrupt bacterial cell membranes and inhibit biofilm formation, enhancing its potential as a natural antibiotic.

*Zingiber officinale*: Ginger’s antimicrobial properties are attributed to its bioactive components such as gingerol and shogaol. These metabolites target microbial cell membranes, inhibiting the growth of pathogenic bacteria and fungi. Gingerol has been particularly effective against respiratory pathogens, highlighting its medicinal significance [27].

*Myristica fragrans*: Nutmeg contains eugenol, a potent antimicrobial compound. Eugenol disrupts bacterial membranes and inhibits toxin production, making it highly effective against foodborne pathogens and other bacterial infections [28].

*Plumbago zeylanica*: This plant’s antimicrobial potential is due to its active compound, plumbagin, which exhibits strong antibacterial and antifungal activities [29]. Plumbagin interferes with bacterial DNA replication and cell wall synthesis, contributing to its broad antimicrobial efficacy.

*Trichosanthes dioica*: *Trichosanthes dioica*, known as the pointed gourd, is a plant with a long history of medicinal use in South Asia. It is known for its various therapeutic properties, including its antimicrobial potential. Research has shown that extracts from *Trichosanthes dioica* possess antibacterial and antifungal activities, making it a valuable plant in the management of infections [30].

*Cinnamomum zeylanicum*: *Cinnamomum zeylanicum*, commonly known as Ceylon cinnamon, is prized for its flavor and medicinal properties. Cinnamon extracts have been extensively studied for their antimicrobial activity, particularly against bacterial and fungal pathogens [31]. The antimicrobial properties of *Cinnamomum zeylanicum* are attributed to its essential oils, which are effective in treating infections and preserving food.

*Elettaria cardamomum: Elettaria cardamomum*, known as green cardamom, is widely used in both culinary and medicinal contexts. α-Pinene and 1,8-Cineole from *Elettaria cardamomum* have been shown to possess antimicrobial properties, particularly against oral pathogens [32,33]. Cardamom is traditionally used to treat digestive issues and respiratory infections, and its antimicrobial activity supports its use in preventing and treating infections.

*Inula racemosa*: *Inula racemosa*, also known as Pushkarmool, is a medicinal plant used in Ayurveda for its anti-inflammatory and antimicrobial properties. Alantolactone from Inula racemosa has demonstrated significant antibacterial activity, particularly against respiratory pathogens [34]. Its use in traditional medicine for treating respiratory ailments is supported by its antimicrobial potential.

*Curcuma longa*: *Curcuma longa*, or turmeric, is a widely recognized medicinal plant known for its anti-inflammatory, antioxidant, and antimicrobial properties. Curcumin, the active compound in turmeric, has been shown to inhibit the growth of a variety of bacterial and fungal pathogens [35]. Turmeric is commonly used in traditional medicine for treating infections and promoting wound healing.

*Piper chaba*: *Piper chaba*, also known as the Javanese long pepper, is used in traditional medicine for its antimicrobial properties. Studies show that *Piper chaba* exhibits antibacterial properties, especially against Gram-positive bacteria, by disrupting microbial membranes and inhibiting efflux pumps [36].

*Carum carvi*: *Carum carvi*, commonly known as caraway, is a spice with medicinal properties, including its antimicrobial activity. Caraway essential is rich in carvone and limonene, which exhibit strong antimicrobial effects [26,37]. These compounds inhibit bacterial and fungal growth, making caraway useful in treating infections and preserving food.

*Terminalia chebula*: *Terminalia chebula*, known as Haritaki, is a key component of Ayurvedic medicine. Its tannins, particularly chebulinic acid, have been shown to inhibit bacterial and fungal pathogens by disrupting their cell walls and preventing their proliferation [38]. *Terminalia chebula* has been shown to inhibit the growth of several bacterial and fungal pathogens, making it an important plant in the treatment of infections.

*Aconitum heterophyllum*: *Aconitum heterophyllum*, or Ativisha, is a medicinal plant used in Ayurveda for its anti-inflammatory and antimicrobial properties. It has been traditionally used to treat fever, infections, and digestive disorders. Research shows that its alkaloids, such as heterophylline, possess significant antibacterial activity, especially against bacterial pathogens responsible for digestive and respiratory infections [39].

*Emblica officinalis*: *Emblica officinalis*, commonly known as Amla or Indian gooseberry, is rich in tannins, flavonoids, and ascorbic acid (vitamin C). Amla extracts have shown effectiveness against a variety of bacterial and fungal pathogens, supporting their traditional use in promoting health and treating infections [40]. Another study reported that Amla-Derived Bionanosilver (Ag NPs) demonstrates excellent antibacterial activity [41].

*Fagonia cretica*: *Fagonia cretica*, commonly known as Dhamasa, is a medicinal plant used in traditional medicine for its antimicrobial properties. Extracts from *Fagonia cretica*, such as flavonoids and tannins, have demonstrated significant antibacterial activity, making them valuable in the treatment of infections [42].

Bharangi (*Clerodendrum serratum*): Bharangi, known as *Clerodendrum serratum*, is a medicinal plant used in Ayurveda for its therapeutic properties, including its antimicrobial effects. It contains terpenoids and flavonoids that exhibit antibacterial and antifungal properties, supporting its use in managing various infections [43].

As shown in Table 5, these plants collectively represent a diverse and potent arsenal of natural antimicrobial agents, with a broad spectrum of activity against various pathogens. Their continued study and application in both traditional and modern medicine hold great promise for the development of new and effective treatments for infectious diseases.

## 3. Discussion

The methodology employed in this study represents a top-down approach, starting with a comprehensive analysis of Ayurvedic formulas—comprising medicinal plants—and progressively narrowing down to the identification of significant bioactive compounds at the plant level. By leveraging cutting-edge machine learning techniques, we identified key metabolites with therapeutic potential, demonstrating the utility of in silico approaches in drug discovery. This approach aligns with prior research emphasizing the importance of computational methods in accelerating the identification of bioactive compounds, especially in traditional medicine systems

Our results reinforce the potential of computational methodologies in uncovering medicinal plants with antibiotic properties. The input dataset included a diverse range of diseases categorized into broader classes, where individual diseases within each class shared certain similarities while retaining unique attributes. Notably, the findings revealed bioactive compounds that exhibited therapeutic efficacy across multiple diseases within a category, reflecting their broad-spectrum potential. This aligns with studies that suggest a high degree of overlap between traditional formulations and their targeted therapeutic effects.

The success of this study lies in the precise identification of medicinal plant candidates and their associated metabolites strongly linked to antibiotic potential. Efficient algorithms enabled the analysis of plant-based formulas to pinpoint compounds likely contributing to the therapeutic efficacy of these traditional remedies. For example, compounds such as gingerol, curcumin, and eugenol identified in this study are well-documented in the literature for their antimicrobial properties, underscoring the validity of our approach.

This work underscores the importance of adopting a systems-level perspective in analyzing traditional medicine. By integrating Ayurvedic knowledge with computational tools, this study bridges the gap between traditional practices and modern drug discovery frameworks. However, the lack of experimental validation remains a limitation. Future work should incorporate empirical testing, such as antimicrobial assays, molecular docking studies, and genetic analyses, to confirm the bioactivity of the identified compounds.

## 4. Materials and Methods

**Figure 2:**
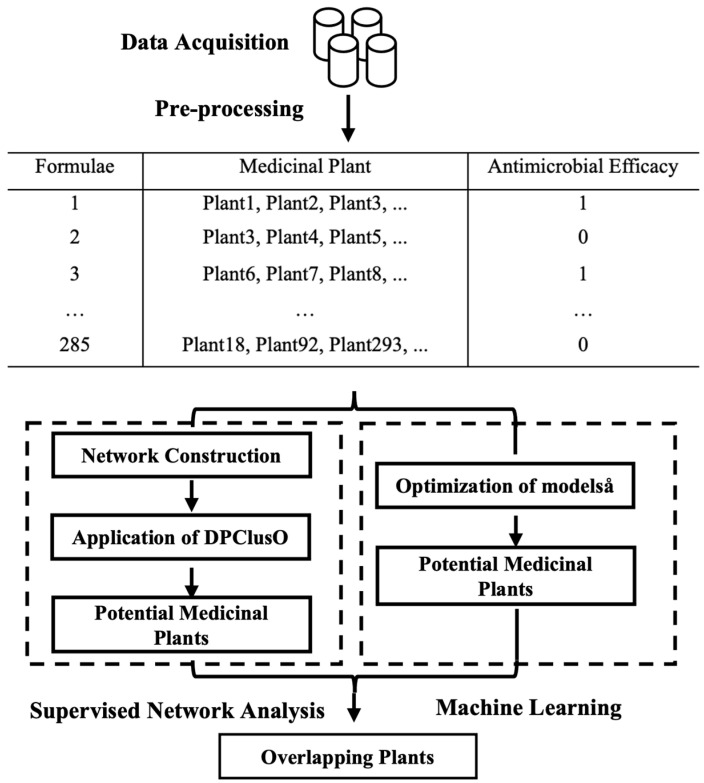
The methodology is structured into four major phases: data acquisition and preprocessing, supervised network clustering, machine learning modeling, and validation.

The methods adopted in the present work are illustrated in the flowchart in Figure 2. The major steps were (1) Data acquisition and preprocessing, (2) Supervised network clustering, (3) Machine Learning approach, and (4) Validation.

### 4.1. Data Acquisition and Preprocessing

In the preliminary phase of this study, we gather comprehensive information on medicinal plants utilized in the composition of Ayurvedic formulae. The initial dataset comprised a collection of Ayurvedic formulae, detailed information on the medicinal plants included in these formulas, and their therapeutic applications. Then, the dataset is structured as tabular data, where each row represents an instance corresponding to a specific Ayurvedic formula. The features of the dataset consist of the medicinal plants included in each formula, along with a class label. The class label is binary, with a value of one indicating that the formula is effective in treating bacterial infections (antibiotic class) and a value of zero indicating otherwise. The dataset utilized in this research has a dimensionality of [285 × 293], encompassing 285 instances and 293 features, with the two distinct class labels representing the effectiveness of the formulas in curing bacterial diseases.

The labeling task involves mapping each herbal formula to cases such as cough, urethritis, typhoid, and similar conditions, categorizing these diseases as bacterial in origin. Herbal formulas effective against bacterial diseases are classified as class 1. However, many conditions, including aches, indigestion, and fever, are not caused by bacteria. This process is challenging due to the absence of a specific database that identifies whether a disease is bacterial. Nonetheless, foundational medical knowledge is applied to assign class labels accurately.

In the phase of data preprocessing, we perform fundamental tasks essential to any data mining process, including checking for and removing missing or redundant data. To further optimize our model, we explored two distinct approaches. The first approach involved reengineering the dataset through dimensionality reduction and the addition of synthetic data. Dimensionality reduction was achieved using Principal Component Analysis (PCA), which was applied to retain the most significant features and streamline the dataset, thereby reducing the model’s complexity. For this study, we retained components that explained at least 95% of the total variance, ensuring that the majority of the dataset’s information was preserved while removing redundant or noisy features. Columns with too many missing values, low variance, or features with little predictive power were filtered out during preprocessing.

The second approach focused on augmenting the dataset by generating new instances of Ayurvedic formulas using the Synthetic Minority Over-sampling Technique (SMOTE). SMOTE is a technique used to handle class imbalance in datasets, where one class (often the positive class) has significantly fewer samples than the others. It works by generating synthetic data points for the minority class. SMOTE creates new instances by interpolating between existing minority class samples, helping the model avoid bias toward the majority class and improving prediction accuracy for underrepresented cases. By creating synthetic examples of the minority classes, SMOTE helped to balance the dataset, thereby potentially improving the model’s performance by mitigating bias towards the majority classes.

### 4.2. Supervised Network Clustering

The DPClusO algorithm was used to cluster Ayurvedic pairs, helping to identify potential medicinal plant–disease relationships, as shown in Figure 2. It is a simple graph clustering algorithm that creates overlapping clusters, ensuring that every node belongs to at least one cluster [45]. The input to DPClusO is an adjacency list of an undirected graph. During each iteration, edges are removed if both connected nodes are part of the newly formed cluster. The process continues until no edges are left in the graph. The algorithm starts by selecting a seed node with the highest weight. If all remaining nodes have a weight of zero, the seed is chosen based on the highest number of connections (degree). A node already in a cluster usually cannot be selected again as a seed. For each neighboring node, the total weight of connections with cluster nodes is calculated, and the node with the highest weight is given priority. If there is a tie, the number of connections is used to decide. If there is still a tie, any one of the tied nodes can be chosen. Priority nodes are added to the cluster in each iteration until the cluster’s density or structure falls below set threshold values.

Numerous studies have extensively utilized this algorithm in network clustering and have demonstrated successful outcomes. To construct the network data, we employed the Minkowski distance as our metric for establishing edges between pairs of Ayurvedic formulas. The goal was to connect formulas whose distance fell below a predefined threshold, specifically a distance value of 1.5. Minkowski distance is a versatile metric for quantifying the distance between two vectors with real-valued components. It generalizes the concepts of Euclidean and Manhattan distances by incorporating an additional parameter known as the order or *p*-value. This parameter allows for the calculation of various distance measurements, each emphasizing different aspects of the data depending on the chosen order. By adjusting the p-value, the Minkowski distance can highlight specific relationships within the data, offering a more nuanced understanding of the proximity between different Ayurvedic formulas. The equation for Minkowski distance, as used in this study, is provided in Equation (1).

(1) $$\begin{array}{c} d_{p}(x,y)={\left(\sum_{i=1}^{n}{\left|x_{i}-y_{i}\right|}^{p}\right)}^{\frac{1}{p}} \end{array},$$

X and Y are Ayurvedic formulae, x_i_ is the binary value of plant-*i*, and y_i_ is the binary value of plant-*i* in the Y Ayurvedic formulae. P is order; if *p* = 1, the equation will be Manhattan distance; if *p* = 2, it will be Euclidian distance.

We map the Ayurvedic formulae to the antibiotics class (class 1) to identify the clusters predominantly associated with antibiotic activity. To accurately determine which clusters are most strongly linked to antibiotic properties, we implemented a voting system. This system assigns each Ayurvedic formula within a cluster to either the antibiotic class or a non-antibiotic class, based on its therapeutic properties. The dominant cluster for antibiotics is then determined using a voting mechanism, where the cluster with the highest proportion of formulas belonging to the antibiotic class is identified as the dominant one. The methodology for this voting system is mathematically represented by Equation (2).

(2) $$\begin{array}{c} Cluster\;score=\frac{number\;of\;Ayurvedic\;Formulae\;belonging\;to\;the\;antibitics\;class}{total\;number\;of\;Ayurvedic\;Formulae\;in\;the\;cluster} \end{array}$$

The resulting clusters of Ayurvedic formulae exhibited high cohesiveness and were separated by a natural boundary, thus facilitating the identification of plant–disease relationships. We performed several parameters, including Function (Filter/Join), density, Cluster Property (CP), Overlapping Coff (OV), and correlation value, and generated multiple outcomes. Based on our analysis, we ultimately selected the network corresponding to a correlation threshold of 0.5 and used CP = 0.4, density = 0.5, and OV = 0.1 to generate the final clusters, and the total cluster we found is 29.

The parameters of the DPClusO algorithm, such as the clustering threshold and density cutoff, were tuned empirically to achieve meaningful groupings of plants. We iteratively adjusted these parameters to balance cluster cohesiveness and biological interpretability, ensuring that the resulting clusters were neither too granular nor overly broad. Preliminary results informed parameter ranges, and manual inspection ensured alignment with domain knowledge.

Although clustering does not inherently follow a train–test split paradigm, we employed a cross-validation-inspired approach by iteratively sampling subsets of the data and re-clustering. This ensured that the identified clusters were stable and not artifacts of specific data configurations.

### 4.3. Learning Models

To identify the most effective machine learning model for our dataset, we conduct an initial evaluation of seven different methods by applying them to the original data. The methods tested, along with their corresponding best accuracy scores, are as follows: Decision Tree Classifier is a tree-based model that splits data iteratively based on feature thresholds to create decision rules. Naïve Bayes Classifier is a probabilistic model based on Bayes’ theorem, which is particularly effective for categorical data. A Gradient Boosting Classifier is an ensemble method that builds models sequentially to correct the errors of previous iterations. K Neighbors Classifier uses proximity-based classification, assigning labels based on the majority class of the nearest neighbors. Logistic Regression models the probability of outcomes using a logistic function and is widely used for binary and multi-class classification. Multilayer Perceptron represents a type of artificial neural network with hidden layers for learning non-linear patterns. The preliminary results indicate that the Random Forest algorithm achieved the highest accuracy, followed by Logistic Regression and Gradient Boosting. To enhance the performance of the random forest model, we performed hyperparameter tuning using a grid search with 5-fold cross-validation. The following parameters, number of trees, maximum depth, minimum samples per leaf, and minimum samples to split were optimized. The best results were achieved with 50 trees, a maximum depth of 15, 5 samples per leaf minimum, and 5 samples to split. The preliminary results revealed that the Random Forest model achieved the highest accuracy at 0.824, which is promising but still not sufficiently accurate for our purposes. Recognizing the need for further improvement, we proceeded with a detailed preprocessing of the dataset. Following preprocessing, we focus on extracting the most important features from the best-performed model. These key features are then used to refine our predictions of medicinal plant-based natural antibiotic candidates.

### 4.4. Validation

This study implemented a rigorous validation process by systematically reviewing previously published scientific literature to verify the efficacy of the identified plants as potential natural antibiotics. The validation process involved a thorough examination of peer-reviewed journals and articles that specifically documented the antibacterial properties of these plants or their natural compounds. This meticulous review focused on evidence demonstrating the ability of the identified plants to inhibit bacterial growth and exhibit significant antibacterial activity, thereby supporting their potential as natural antibiotics.

## 5. Conclusions

In this paper, we employed a combination of supervised network clustering and machine learning techniques to identify potential plant-based natural antibiotics from Ayurvedic formulations. The integration of data preprocessing, dimensionality reduction, and synthetic data augmentation enhanced the model’s accuracy. The Random Forest model, identified as the most effective, highlighted key medicinal plants with significant antibacterial properties. Rigorous validation through existing scientific literature confirmed the potential of these plants as natural antibiotics, offering valuable insights for the development of new therapeutic agents to combat antibiotic-resistant bacteria.

## Figures and Tables

**Table 1:** Frequency distribution of Medicinal Herb Features in Ayurvedic Formulae Dataset.

No.	Medicinal Plant	Frequency
1	*Piper longum*	102
2	*Zingiber officinale*	93
3	*Emblica officinalis*	74
4	*Piper nigrum*	69
5	*Terminalia chebula*	56
6	*Myristica fragrans*	54
7	*Cyperus rotundus*	53
8	*Tenninalia chebula*	50
9	*Terminalia bellcrica*	37
10	*Piper chaba*	36
11	*Plumbago zeylanica*	34
12	*Syzygium aromaticum*	32
13	*Coriandrum sativum*	32
14	*Cinnamomum zeylanicum*	30
15	*Elettaria cardamomum*	30
16	*Saussurea hypoleuca*	30
17	*Aconitum ferox*	29
18	*Aloe barbadensis*	29
19	*Tinospora cordifolia*	29
20	*Nigella sativa*	29
21	*Carum curvi*	28
22	*Cinnamomum tamala*	28
23	*Glycyrrhiza glabra*	27
24	*Pterocarpus santalinus*	27
25	*Berberis aristata*	26
26	*Embelia ribes*	25
27	*Cedrus deodara*	24
28	*Acorus calamus*	24
29	*Tribulus terresrris*	23
30	*Curcuma longa*	23
31	*Aegle marmelos*	22
32	*Trachyspermum ammi*	22
33	*Sida cordifolia*	22
34	*Aconitum heterophyllum*	21
35	*Operculina turpethum*	21
36	*Mesua ferrea*	20
37	*Rhus succedanea*	19
38	*Curcuma zedoaria*	19
39	*Hollerrhena antidysenterica*	18
40	*Picrorhiza kurroa*	18

**Table 2:** Accuracy of Machine Learning Methods.

Machine Learning Models	Filtering	SMOTE	Filtering + SMOTE
Decision Tree Classifier	0.673 ± 0.025	0.648 ± 0.015	0.635 ± 0.010
Naïve Bayes Classifier	0.626 ± 0.013	0.675 ± 0.028	0.648 ± 0.017
Gradient Boosting Classifier	0.748 ± 0.014	0.748 ± 0.011	0.775 ± 0.026
K-Neighbors Classifier	0.755 ± 0.009	0.709 ± 0.017	0.746 ± 0.012
Logistic Regression	0.790 ± 0.016	0.755 ± 0.008	0.797 ± 0.015
Multi-layer Perceptron	0.671 ± 0.020	0.752 ± 0.014	0.748 ± 0.025
Random Forest	0.727 ± 0.022	0.774 ± 0.014	0.824 ± 0.018

**Table 3:** Plants prediction from supervised network clustering.

No.	Medicinal Plant	Ayurvedic Formulae
1	*Zingiber officinale*	6
2	*Cyperus rotundus*	5
3	*Piper longum*	5
4	*Nigella sativa*	4
5	*Rhus succedanea*	4
6	*Tinospora cordifolia*	4
7	*Terminalia chebula*	4
8	*Bharangi—Clerodendrum*	3
9	*Carum curvi*	3
10	*Cedrus deodara*	3
11	*Coriandrum sativum*	3
12	*Emblica officinalis*	3
13	*Myrica nagi/Myrica sapida*	3
14	*Piper nigrum*	3
15	*Saussurea hypoleuca*	3
16	*Fagonia cretica*	3
17	*Picrorhiza kurroa*	3
18	*Cinnamomum tamala*	2
19	*Cinnamomum zeylanicum*	2
20	*Croton polyandrum*	2
21	*Elettaria cardamomum*	2
22	*Glycyrrhiza glabra*	2
23	*Myristica fragrans*	2
24	*Piper chaba*	2
25	*Plumbago zeylanica*	2
26	*Syzygium aromaticum*	2
27	*Trachyspermum ammi carum copticum*	2
28	*Trianthema portulacastrum*	2
29	*Tribulus terrestris*	2
30	*Uraria lagopoides*	2
31	*Aegle marmelos*	2
32	*Solanum indicum*	2
33	*Aconitum heterophyllum*	2
34	*Cissampelos pareira*	2
35	*Azadirachta indica*	2
36	*Curcuma longa*	2
37	*Trichosanthes dioica*	2
38	*Inula racemosa*	2
39	*Nigella sativa*	2

**Table 4:** Medicinal plant weights from machine learning model.

No.	Medicinal Plant	Weight
1	*Cyperus rotundus*	0.03109277
2	*Piper longum*	0.0261781
3	*Aconitum ferox*	0.02305289
4	*Sida cordifolia*	0.01956573
5	*Piper nigrum*	0.01927294
6	*Zingiber officinale*	0.01859687
7	*Myristica fragrans*	0.01832107
8	*Plumbago zeylanica*	0.01659803
9	*Acacia leucophloea*	0.01593949
10	*Terminalia chebula*	0.01574221
11	*Trichosanthes dioica*	0.01567844
12	*Cinnamomum zeylanicum*	0.01544429
13	*Bambusa bambos*	0.01483098
14	*Elettaria cardamomum*	0.0143906
15	*Inula racemosa*	0.0140497
16	*Curcuma longa*	0.01371762
17	*Piper chaba*	0.0136643
18	*Punica granatum*	0.01346506
19	*Carum curvi*	0.01325254
20	*Femia foetida*	0.01210255
21	*Tenninalia chebula*	0.01149938
22	*Aconitum heterophyllum*	0.0111556
23	*Emblica officinalis*	0.01114156
24	*Adhatoda vasica*	0.01106956
25	*Fagonia cretica*	0.01092521
26	*Berberies aristata*	0.01090131
27	*Berberis aristata*	0.0107864
28	*Bharangi—Clerodendrum*	0.01015191
29	*Syzygium aromaticum*	0.01012223
30	*Operculina turpethum*	0.01011386
31	*Terminalia bellcrica*	0.01009841
32	*Hollerrhena antidysentrica*	0.01003895

**Table 5:** Academic recordings as evidence for the overlapping medicinal plants using supervised network analysis and machine learning approaches.

No.	Medicinal Plant	Metabolite	Antimicrobial Property	Reference
1	*Piper longum*	isolates	Antibacterial	[44]
2	*Piper nigrum*	β-Caryophyllene (C15H24), limonene (C10H16)	Antibacterial, Antifungal	[25,26]
3	*Zingiber officinale*	Gingerol (C17H26O4)	Antibacterial	[27]
4	*Myristica fragrans*	Eugenol (C10H12O2)	Antibacterial, Antifungal	[28]
5	*Plumbago zeylanica*	plumbagin (C11H8O3)	Antibacterial	[29]
6	*Trichosanthes dioica*	isolates	Antibacterial	[30]
7	*Cinnamomum zeylanicum*	isolates	Antibacterial, Antifungal	[31]
8	*Elettaria cardamomum*	α-Pinene (C10H16), 1,8-Cineole (C10H18O)	Antibacterial, Antifungal	[32,33]
9	*Inula racemosa*	Alantolactone (C15H20O2)	Antibacterial	[34]
10	*Curcuma longa*	Curcumin (C21H20O6)	Antibacterial, Antioxidant	[35]
11	*Piper chaba*	isolates	Antibacterial, Antiviral	[36]
12	*Carum carvi*	carvone (C10H14O), limonene (C10H16)	Antibacterial	[26,37]
13	*Terminalia chebula*	isolates	Antibacterial, Antiviral	[38]
14	*Aconitum heterophyllum*	heterophylline (C22H26N2O4)	Antibacterial	[39]
15	*Emblica officinalis*	isolates, Ag NPs	Antibacterial, Antioxidant	[40,41]
16	*Fagonia cretica*	isolates	Antibacterial	[42]
17	*Bharangi*	isolates	Antibacterial	[43]

## Data Availability

Data will be made available on request.
